# Prevalence of insomnia and its association with quality of life in caregivers of psychiatric inpatients during the COVID-19 pandemic: a network analysis

**DOI:** 10.1186/s12888-023-05194-w

**Published:** 2023-11-14

**Authors:** Pan Chen, Yan-Jie Zhao, Feng-Rong An, Xiao-Hong Li, Mei Ieng Lam, Ka-In Lok, Yue-Ying Wang, Jia-Xin Li, Zhaohui Su, Teris Cheung, Gabor S. Ungvari, Chee H. Ng, Qinge Zhang, Yu-Tao Xiang

**Affiliations:** 1 Unit of Psychiatry, Department of Public Health and Medicinal Administration, & Institute of Translational Medicine, Faculty of Health Sciences, University of Macau, Macao SAR, China; 2 Centre for Cognitive and Brain Sciences, University of Macau, Macao SAR, China; 3 Beijing Key Laboratory of Mental Disorders, National Clinical Research Center for Mental Disorders & National Center for Mental Disorders, Beijing Anding Hospital, Capital Medical University, Beijing, China; 4 Beijing Huilongguan Hospital, Peking University Huilongguan Clinical Medical School, Beijing, China; 5 Kiang Wu Nursing College of Macau, Macao SAR, China; 6 Faculty of Health Sciences and Sports, Macao Polytechnic University, Macao SAR, China; 7 School of Public Health, Southeast University, Nanjing, China; 8 School of Nursing, Hong Kong Polytechnic University, Hong Kong SAR, China; 9 University of Notre Dame Australia, Fremantle, Australia; 10 Division of Psychiatry, School of Medicine, University of Western Australia, Perth, Australia; 11 Department of Psychiatry, The Melbourne Clinic and St Vincent’s Hospital, University of Melbourne, Richmond, VIC Australia

**Keywords:** Insomnia, Quality of life, COVID-19, Caregivers, Network analysis

## Abstract

**Background:**

Studies on sleep problems among caregivers of psychiatric patients, especially during the COVID-19 pandemic, are limited. This study examined the prevalence and correlates of insomnia symptoms (insomnia hereafter) among caregivers of psychiatric inpatients during the COVID-19 pandemic as well as the association with quality of life (QoL) from a network analysis perspective.

**Methods:**

A multi-center cross-sectional study was conducted on caregivers of inpatients across seven tertiary psychiatric hospitals and psychiatric units of general hospitals. Network analysis explored the structure of insomnia using the R program. The centrality index of “Expected influence” was used to identify central symptoms in the network, and the “flow” function was adopted to identify specific symptoms that were directly associated with QoL.

**Results:**

A total of 1,101 caregivers were included. The overall prevalence of insomnia was 18.9% (n = 208; 95% CI = 16.7–21.3%). Severe depressive (OR = 1.185; P < 0.001) and anxiety symptoms (OR = 1.099; P = 0.003), and severe fatigue (OR = 1.320; P < 0.001) were associated with more severe insomnia. The most central nodes included ISI2 (“Sleep maintenance”), ISI7 (“Distress caused by the sleep difficulties”) and ISI1 (“Severity of sleep onset”), while “Sleep dissatisfaction” (ISI4), “Distress caused by the sleep difficulties” (ISI7) and “Interference with daytime functioning” (ISI5) had the strongest negative associations with QoL.

**Conclusion:**

The insomnia prevalence was high among caregivers of psychiatric inpatients during the COVID-19 pandemic, particularly in those with depression, anxiety and fatigue. Considering the negative impact of insomnia on QoL, effective interventions that address insomnia and alteration of sleep dissatisfaction should be developed.

**Supplementary Information:**

The online version contains supplementary material available at 10.1186/s12888-023-05194-w.

## Introduction

Caregivers are defined as “primary persons who generally provide the majority of care and support to patients”, and are an extension of patients’ hospital care support system [1]. They undertake the primary task of caring for patients and play a valuable caregiving role in managing the lives of patients during their hospital stay [2]. To some extent, they are also viewed as effective legal decision-makers on medical issues for patients they are responsible for [3]. They are a hidden workforce that alleviate the burden of professional care and the costs of social care systems [2]. Caregiving burden is commonly divided into objective and subjective burdens [4]. Objective burden refers to the impact of the caregiving tasks on the caregivers’ household activities, economic resources, health and leisure activities, while subjective burden refers to the personal distress suffered as a result of giving care such as feelings of loss, shame and anger [4]. Previous studies have found a high burden in caregivers of psychiatric patients with schizophrenia (SCZ), bipolar disorder (BP), and major depressive disorder (MDD) [5], which could affect the caregivers’ quality of life (QoL) and family function [6]. Additionally, illness factors were significantly associated with a high burden in caregivers, including duration of mental illness, severity of symptoms and level of dysfunction [1, 7]. Caring for patients with severe psychiatric disorders could compromise caregivers’ mental health and result in psychological distress, anxiety, depression, insomnia and post-traumatic stress disorder [8]. Psychiatric patients often suffer from anxiety, depression, stress, insomnia, impulsivity and even suicidal ideation [9], which may contribute to their caregivers’ distress.

Insomnia is a common problem in patients with psychiatric disorders [10], which can lead to an exacerbation of a pre-existing psychiatric disorder or be a reaction to psychological distress [11]. As such, caregiving burden can directly result in sleep disturbances [12]. A previous study found that family caregivers were more likely to report insomnia than non-caregivers (46% vs. 37%) [13]. Other studies showed that caregivers had a high prevalence of sleep disturbance, for example, 50–70% of caregivers of dementia patients [14] and 40% of caregivers of cancer patients [15] experienced insomnia. Common insomnia complaints in caregivers for patients with cancers included short sleep duration, nocturnal awakenings, waking after the onset of sleep, and daytime dysfunction [15]. However, the features of insomnia among caregivers of patients with psychiatric disorders are not clear.

Insomnia in caregivers was found to be associated with various factors. Most studies found that female caregivers were more likely to experience sleep disturbance [16]. Insomnia was commonly associated with psychiatric problems among caregivers compared to non-caregivers, especially depression and anxiety disorder; for instance, 44% of caregivers of bipolar patients had a diagnosis of anxiety disorder [17]. In addition, fatigue was associated with an increased risk of sleep problems [18].

The occurrence of mental health problems in many populations greatly increased during the COVID-19 pandemic [19]. Poor psychological status was more common among caregivers of patients with physical or intellectual disabilities [20]. Further, a comparative study found that the prevalence of insomnia among caregivers of mentally ill patients or physically disabled patients during the COVID-19 lockdown was higher than non-caregivers (69.9% vs. 44.7%) [21]. However, studies on insomnia among caregivers for psychiatric patients during the COVID-19 pandemic have been limited. Insomnia could result in adverse health outcomes, such as poor mental health status, daily functioning and caregiving quality, which in turn could worsen the QoL of caregivers [14]. Although the association between insomnia and QoL has been documented in many studies [22, 23], the relationships between individual insomnia symptoms and QoL among the caregivers of psychiatric patients are still unclear.

Traditionally, the severity of psychiatric disorders or syndromes are measured and analyzed using the sum score of the assessment tools; for example, insomnia is measured by the Insomnia Severity Index (ISI) [24]. However, traditional approaches obscure the potential differences and inter-relationships between different symptoms of a psychiatric disorder or syndrome. As an emerging novel approach to examine the complex and dynamic interactions among symptoms, network analysis can overcome this limitation, based on the premise that a particular psychiatric syndrome is viewed as an interacting cluster of symptoms, with different strength and nature of associations between the psychiatric symptoms [25]. Network analysis can identify the most influential symptoms (central nodes) and the strongest relationships (edges) between different symptoms. In a network model, the central symptoms have the strong connections with other symptoms, which play an important role in the causation and/or maintenance of the psychiatric disorder or syndrome by activating or deactivation other symptoms [25]. Thus, the identification of central symptoms is clinically important for developing effective targeted strategies or interventions to treat psychiatric disorders or syndromes.

To date, network analysis of insomnia symptoms has already been applied to general populations [26, 27] and mental health professionals [28]. However, no network analysis studies on insomnia symptoms among caregivers of psychiatric inpatients have been published. To fill this gap, this study examined the prevalence and correlates of insomnia in caregivers of psychiatric inpatients, constructed a network model of insomnia symptoms, and evaluated the relationships between insomnia symptoms and QoL.

## Methods

### Participants and procedures

**Fig. 1:**
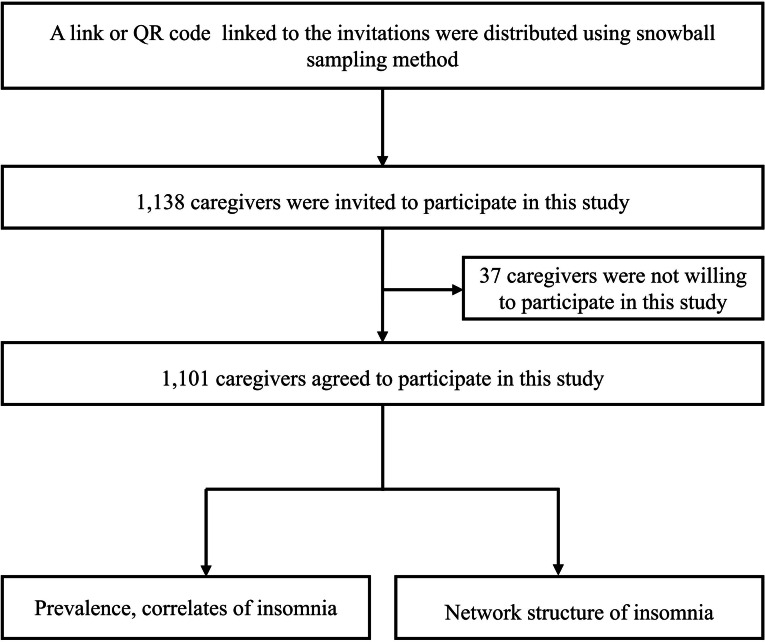
Flow chart of study design

A multi-center cross-sectional study was conducted between May 24, 2020 and January 18, 2021 among caregivers of psychiatric inpatients using a consecutive sampling method across seven tertiary psychiatric hospitals and psychiatric units of general hospitals located in Liaoning, Shandong, Beijing, Chongqing, Hunan, Guangdong, and Xinjiang in China (Fig. 1). Due to the risks of transmission of COVID-19, face-to-face assessments were not adopted. Data collection was performed using WeChat program during the period of the patients’ hospitalization. All people including caregivers needed to report their health status using WeChat when they entered hospitals during the pandemic; therefore, they were all presumably WeChat users. In China, caregivers might include family members, relatives, close friends of patients or designated hospital employees. All caregivers who visited the patients in the participating hospitals during the study period were consecutively invited by scanning a QR code linked to the invitations. The inclusion criteria were as follows: (1) aged 18 years or above; (2) caregivers of psychiatric patients hospitalized in the participating hospital during the study period such as spouses, parents, offsprings, and other kins or friends; (3) no current episodes of major psychiatric disorders and not co-hospitalized with patients; and (4) able to understand the purpose of the survey and complete the assessment. All participants who provided electronic written informed consent on a voluntary and confidential basis were included in this study. Approval of the study was granted by the Institutional Review Board of Beijing Anding hospital and respective hospitals.

### Measures

Basic socio-demographic and clinical characteristics of participants were collected, including age, gender, marital status, education level, employment status, living areas, presence of any major physical diseases, perceived financial status, frequency of social media use, and difficulty in accessing mental health services during the pandemic. Additionally, the patients’ principal psychiatric diagnoses i.e., MDD, BP, SCZ, or others, and medication adherence were recorded.

The validated Chinse version of the Insomnia Severity Index (ISI) questionnaire [24] was used to assess insomnia symptoms in the participants within the past week, which included seven domains: (1) severity of sleep onset problem; (2) sleep maintenance problem; (3) early morning wakening problem; (4) sleep dissatisfaction; (5) interference with daytime function; (6) noticeability of sleep problems by others, and (7) distress caused by the sleep difficulties. Each item was scored on a scale from “0” (no problem) to “4” (very severe problem). The total score ranged from 0 to 28, with a higher score reflecting more severe insomnia symptoms. The cut-off value of 8 was considered as having insomnia symptoms, which can be further categorized into subthreshold insomnia (8–14 points), moderate clinical insomnia (15–21 points), and severe clinical insomnia (22–28 points) [29].

Depressive and anxiety symptoms were evaluated using the validated Chinese version of the Patient Health Questionnaire (PHQ-9) [30] and Generalized Anxiety Disorder scale (GAD-7) [31], respectively. Each item of the two scales was rated on a 4-point Likert scale from “0” (not at all) to “3” (nearly every day). The total scores of the PHQ-9 (0 to 27) and GAD-7 (0 to 21) were the sum of all the item scores, respectively. Subjective fatigue was assessed using a numeric rating scale (NRS), which ranged from “0” (no fatigue) to “10” (worst fatigue imaginable) [32]. The global QoL of caregivers of psychiatric inpatients was assessed using the total score of the first two items of the World Health Organization Quality of Life-brief version (WHOQOL-BREF) [33], with a higher total score indicating higher QoL.

### Data analysis

#### Univariate and multivariate analyses

Univariate and multivariate analyses were performed using SPSS version 26.0 (SPSS Inc., Chicago, Illinois, USA). Shapiro-Wilk tests and Q-Q plots were used to test the normality of the distributions for continuous variables. Participants were divided into insomnia and non-insomnia groups. Independent sample t-tests, Mann-Whitney U tests, and Chi-square tests were conducted as appropriately to compare the differences in demographic and clinical characteristics between the two groups. Analysis of covariance (ANCOVA) was used to compare QoL between insomnia and non-insomnia groups after controlling for variables that showed significant differences in univariate analyses. Binary logistic regression analysis was performed to examine independent correlates of insomnia, with insomnia as the dependent variable, and those with significant group differences in univariate analyses were entered as independent variables. The statistical significance level was set at P < 0.05 (two-tailed) for all analyses.

#### Network structure

Network analysis was conducted using the R program (4.2.2 version) [34]. Symptoms were presented as nodes, and partial correlations between symptoms were presented as edges. The thickness of the edges reflected the strength of the correlations between the nodes, with green edges indicating positive correlations and red indicating negative correlations. As some of the ISI item scores did not follow normal distribution, nonparametric correlations were calculated via nonparanormal transformations. The network structure of insomnia symptoms was constructed using the Graphical Gaussian Model (GGM)following nonparanormal transformation of the data. The graphic least absolute shrinkage and selection operator (LASSO) in combination with Extended Bayesian Information Criterion (EBIC) were used to estimate a regularized GGM [35]. Considering the potential effects of age on sleep [36], the network model after controlling for age was also constructed. Network estimation was conducted using the ‘bootnet’ package [37], visualized using the ‘qgraph’ package [38] and optimized using the “ggplot2” package [39]. Additionally, the ‘flow’ function in the R package ‘qgraph’ was adopted to identify specific insomnia symptoms that were directly associated with QoL [38].

For centrality index, the expected influence (EI) index of each node was computed using “bootnet” package to determine the most central symptoms in the network model [40]. The predictability was calculated using the “mgm” package [41], reflecting the variance of a node that could be explained by its neighbor nodes in the model. To compare the original network model with the one after controlling for age, Spearman’s rank correlation coefficient was calculated to test the correlation of EI between both networks [42].

The stability and accuracy of the network model were evaluated using the “bootnet” package [35]. The correlation stability coefficient (CS-C) was used to evaluate the stability of the network model; a value of above 0.25 was regarded as acceptable stability although 0.5 was preferable [35]. Non-parametric bootstrapped 95% confidence intervals (CI) of edge weights were estimated to evaluate the network accuracy, with a narrower CI indicating a more reliable network. A nonparametric bootstrapped difference test was performed to examine whether two edge-weights significantly differed from one-another.

The Network Comparison Test (NCT) was used to compare the network structures of insomnia symptoms between genders using the “Network Comparison Test” package [43]. The indices of network structure invariance, global strength invariance, and edges or nodes centrality invariance were used to evaluate the results of comparisons [43].

## Result

### Participant characteristics

Of the 1,138 caregivers invited, 1,101 agreed to participate in the study and completed the assessment, giving a participation rate of 97.3%. Demographic and clinical characteristics of the participants are shown in Table 1. The mean age of participants was 43.06 (SD = 11.6) years and 40.1% (n = 442) were male. Over half of the participants had an education level of senior secondary school and above (n = 677; 61.5%). More than 80% of participants were married (n = 903; 82.0%) and employed (n = 894; 81.2%).

**Table 1:** Demographic and clinical characteristics of the study sample (N = 1,101)

Variables	Total (N = 1,101)		Without insomnia (N = 893)		With insomnia (N = 208)		Univariable analysis		
	n	%	n	%	n	%	${\chi }^{2}$	*df*	*p*
Male	442	40.1	345	38.6	97	46.6	4.494	1	**0.034**
Married	903	82.0	740	82.9	163	78.4	2.318	1	0.128
Employed	894	81.2	726	91.3	168	80.8	0.031	1	0.860
Senior secondary school and above	677	61.5	547	61.3	130	62.5	0.111	1	0.739
Living in rural areas	468	42.5	387	43.3	81	38.9	1.333	1	0.248
Presence of major physical diseases	52	4.7	35	3.9	17	8.2	6.784	1	**0.009**
Perceived financial status							16.872	2	**< 0.001**
Poor	234	21.3	172	19.3	62	29.8			
Fair	760	69	623	69.8	137	65.9			
Good	107	9.7	98	11.0	9	4.3			
Frequency of social media use							3.419	2	0.181
No or minimal	85	7.7	72	8.1	13	6.3			
Sometimes	356	32.3	278	31.1	78	37.5			
Often	660	59.9	543	60.8	117	56.3			
Difficulty in accessing mental health service during the pandemic	327	29.7	245	27.4	82	39.4	11.611	1	**0.001**
	Mean	*SD*	Mean	*SD*	Mean	*SD*	*t/Z*	*df*	*p*
Age (years)	43.06	11.641	42.99	11.337	43.34	12.892	-0.360	286.122	0.719
PHQ-9 total	3.95	5.654	2.33	3.842	10.91	6.799	-18.395	---*	**< 0.001**
GAD-7 total	3.04	4.609	1.79	3.155	8.41	5.856	-17.005	---*	**< 0.001**
Fatigue	3.07	2.482	2.55	2.239	5.31	2.224	-14.028	---*	**< 0.001**
Global quality of life	6.56	1.682	6.90	1.554	5.10	1.409	15.328	1099	**< 0.001**
**Patients’ information**									
	n	%	n	%	n	%	${\chi }^{2}$	*df*	*p*
Principal psychiatric diagnosis							7.751	3	0.051
Major depressive disorder	400	36.3	317	35.5	83	39.9			
Bipolar disorder	162	14.7	123	13.8	39	18.8			
Schizophrenia	222	20.2	191	21.4	31	14.9			
Others	317	28.8	262	29.3	55	26.4			
Good treatment adherence during the pandemic	775	70.4	634	71.0	141	67.8	0.833	1	0.361

Bolded values: <0.05; Abbreviation: df: degree of freedom; PHQ-9: Patient Health Questionnaire-9 items; GAD-7: Generalized Anxiety Disorder-7 items; SD: standard deviation. * Mann-Whitney U test.

### Prevalence and correlates of insomnia

The overall prevalence of insomnia (ISI total score ≥ 8) among caregivers of psychiatric inpatients during the COVID-19 pandemic was 18.9% (n = 208; 95% CI = 16.7–21.3%); specifically, 157 (14.3%, 95% CI = 12.3–16.5%) reported subthreshold insomnia (ISI total score: 8–14), 44 (4.0%, 95% CI = 3.0-5.3%) reported moderate clinical insomnia (ISI total score: 15–21), and 7 (0.6%, 95% CI = 0.3–1.3%) reported severe clinical insomnia (ISI total score: 22–28).

Compared with the non-insomnia group, caregivers with insomnia were more likely to be male (P = 0.034) and had major physical diseases (P = 0.009), difficulty in visiting mental health services during the pandemic (P = 0.001), and perceived poor financial status (P < 0.001). Moreover, participants with insomnia were more likely to report a higher score on PHQ-9 (P < 0.001), GAD-7 (P < 0.001), and fatigue (P < 0.001), but a lower score on QoL (P < 0.001) (Table 1). ANCOVA results indicated that caregivers with insomnia had a poorer global QoL (F _(1,101)_ = 82.276, P < 0.001) compared to those without insomnia. Binary logistic regression analysis revealed that more severe depressive (OR = 1.185; P < 0.001) and anxiety symptoms (OR = 1.099; P = 0.003) and fatigue (OR = 1.320; P < 0.001) were significantly associated with higher risk of insomnia (Table 2).

**Table 2:** Independent correlates of insomnia among caregivers of psychiatric inpatient during the COVID-19 pandemic (N = 1,101)

Variables	Multiple logistic regression analysis*		
	*p*	*OR*	95% *CI*
Male	0.093	1.410	0.945–2.106
Presence of major physical diseases	0.881	1.070	0.442–2.589
Perceived financial status (poor vs. fair)	0.433	0.827	0.515–1.328
Perceived financial status (poor vs. good)	0.062	0.411	0.162–1.044
Difficulty in accessing mental health service during the pandemic	0.420	0.841	0.551–1.282
PHQ-9 total	**< 0.001**	1.185	1.122–1.251
GAD-7 total	**0.003**	1.099	1.032–1.171
Fatigue	**< 0.001**	1.320	1.206–1.445

Bolded values: <0.05; Abbreviations: *CI*: confidence interval; *OR*: odds ratio; *adjusted for study site as a covariate variable

### Network structure of insomnia symptoms

**Fig. 2:**
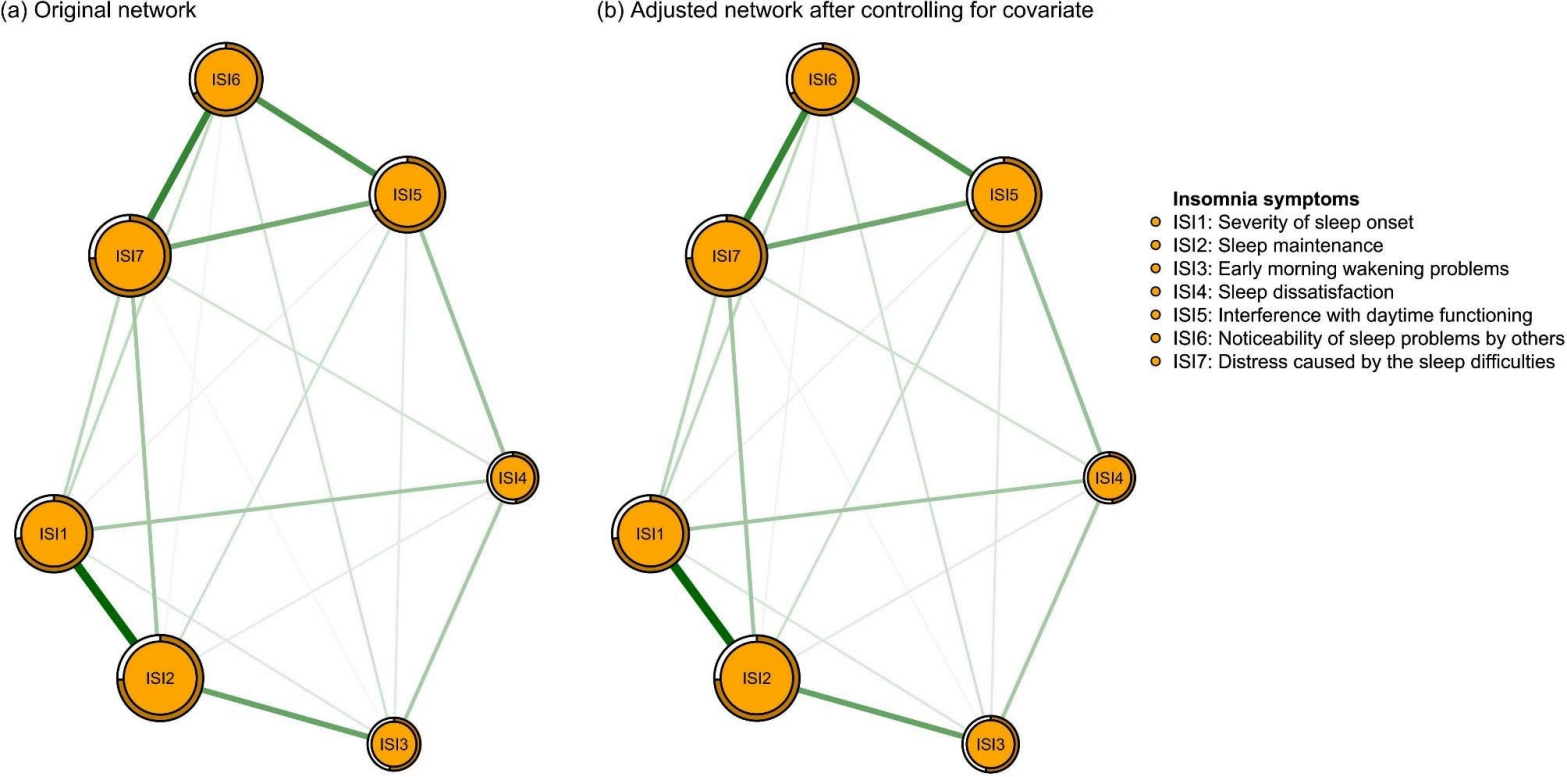
Network structure of insomnia symptoms among caregivers of psychiatric inpatients

**Fig. 3:**
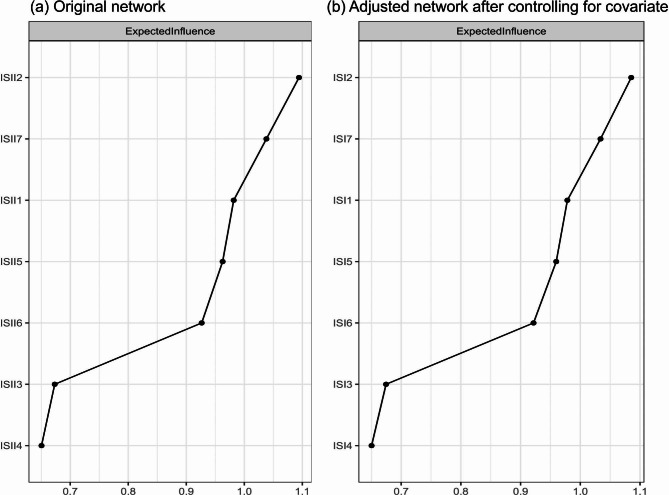
Network centrality of insomnia symptoms among caregivers of psychiatric inpatients

**Fig. 4:**
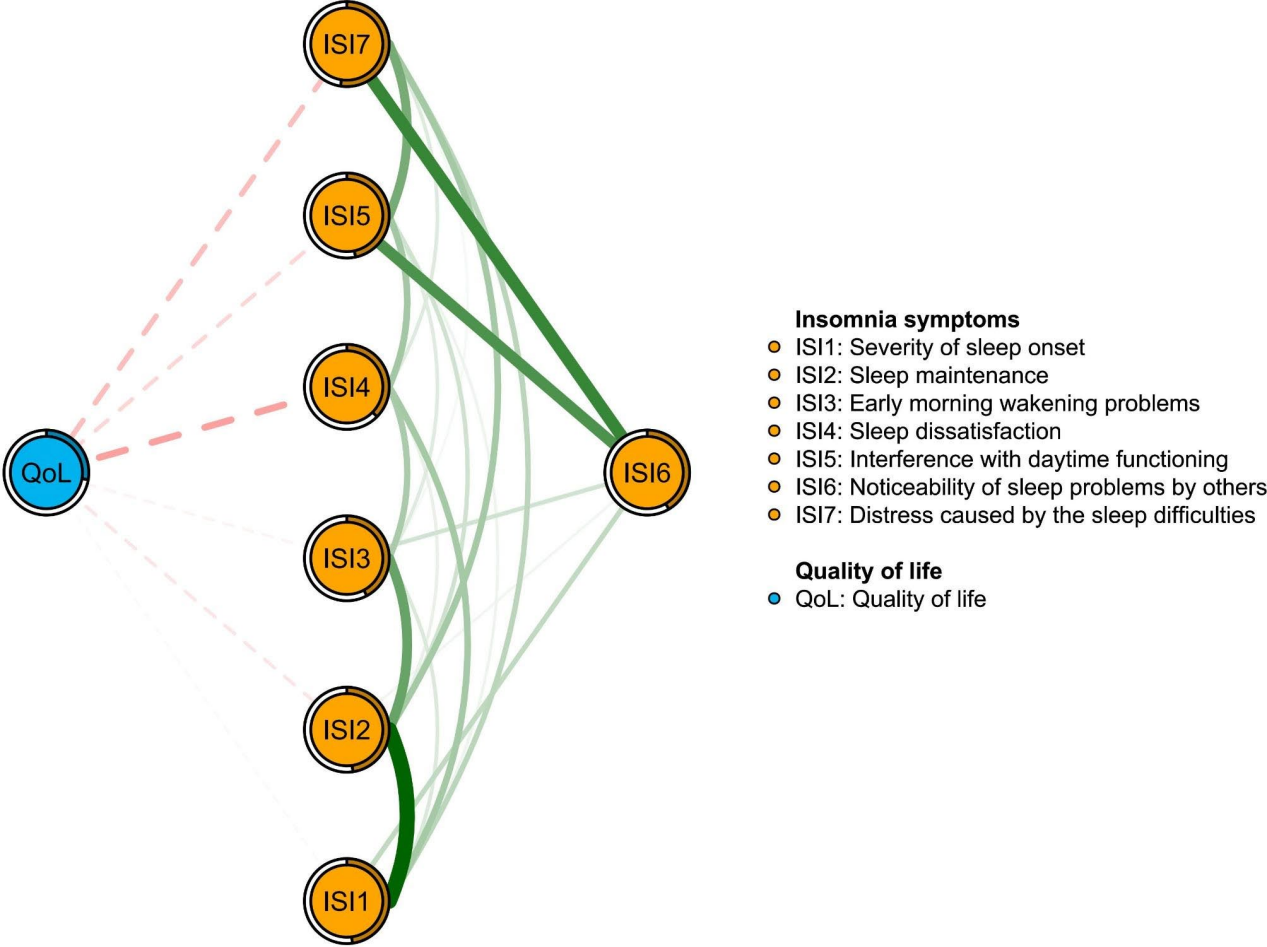
Flow network of quality of life

Figure 2 presents the original network structure of insomnia symptoms and the adjusted network model after controlling for age among caregivers of psychiatric inpatients during the COVID-19 pandemic. Figure 3 shows that the top three nodes with the highest centrality were ISI2 (“Sleep maintenance”), ISI7 (“Distress caused by the sleep difficulties”) and ISI1 (“Severity of sleep onset”) in both networks. The mean predictability in the original insomnia network was 0.654, indicating that there was an average of 65.4% of the variance in each node that could be accounted for by its neighboring nodes in the model. Table S1 shows the descriptive information of insomnia symptoms in the original and adjusted network models. The similarity tests showed that the EI generated from the original and adjusted insomnia network models were highly correlated (r = 0.99, P < 0.01). Furthermore, the NCT results did not show any significant differences in the network structure (M = 0.227, P = 0.08), global strength (S = 0.052, P = 0.677) and nodes centrality test (C=-0.032, P = 0.09) between the male and female network models of insomnia symptoms. The flow network (Fig. 4) shows that the edge between ISI4 (“Sleep dissatisfaction”; average edge weight=-0.18) and QoL is the thickest one marked in red, indicating that ISI4 (“Sleep dissatisfaction”) had the strongest negative association with QoL, followed by the ISI7 (“Distress caused by the sleep difficulties”; average edge weight=-0.12) and ISI5 (“Interference with daytime functioning”; average edge weight=-0.07). The details of the edge weights of these symptoms are shown in Table S2. Figure S1 presents the network stability based on the case-dropping bootstrap procedure. The CS-C for EI was 0.75, suggesting sufficient stability. For network accuracy, a narrow range was shown in the bootstrap 95% CIs for the estimated edge weights (Figure S2), and most of them were non-zero, indicating that most edges were stable and accurate. The bootstrapped difference tests for edge weights in Figure S3 show that most comparisons among edge weights were statistically significant, indicating a reliable network model.

## Discussion

To the best of our knowledge, this was the first study to explore the prevalence and network structure of insomnia among caregivers of psychiatric inpatients during the COVID-19 pandemic. Of the participants, 18.9% suffered from insomnia (ISI total score ≥ 8; 95% CI: 16.7–21.3%), which is higher than the corresponding figure in the general population reported in previous studies in China both before (15.0%; 95%CI: 12.1–18.5%) [44] and during the COVID-19 pandemic (16.5%; 95% CI: 8.4–29.7%) [45]. However, another population survey conducted in 125 cities in mainland China during COVID-19 pandemic found a higher rate (24.7%) than our study [46]. There might be common COVID-19 related factors associated with insomnia among the caregivers, such as the fear of contracting the infection, being quarantined, and financial burden [45]. Psychiatric inpatients were at higher risk of infection compared to the general population due to several reasons: psychiatric hospitals have closed wards with restricted space and poor quality of air; the use of antipsychotics is associated with reduced ability to eliminate pathogenic bacteria in the respiratory tract; and psychiatric inpatients who are mentally unwell often lack the capacity to practice self-protection measures [47]. Additionally, the strict infection prevention policies of hospitals to minimize hospital transmission substantially restricted the caregivers’ access to patients or ability to provide caregiving, which might aggravate their anxiety and uncertainty about the patients [47]. Interestingly, the insomnia rate in our study was much lower than the findings using the same assessment scale and cutoff value in caregivers living with family members with disabilities in the same household (69.9%) [21], hospice family caregivers (49.1%) [48] and caregivers of children with kidney diseases (38.5%) [49]. It is likely that the different types and severity of diseases were associated with different levels of the distress among the caregivers [50].

In both original and adjusted network models of insomnia symptoms, “Sleep maintenance” (ISI2), “Distress caused by the sleep difficulties” (ISI7), and “Severity of sleep onset” (ISI1) were the most influential symptoms. As both network models were highly similar and no gender differences in the network model of insomnia symptoms were found, this suggests that the insomnia symptoms network was stable and not affected by age or gender. Nocturnal symptoms (e.g., ISI1, ISI2, ISI3) appeared to be dominant in this network model. Inpatients with psychiatric disorders often have sleep problems and even an increased risk of suicide at night time [51], and as such, due to increased vigilance, this would more likely cause difficulty for their caregivers in getting to sleep or maintaining sleep at night [52]. Previous studies found that caring for patients at night was a major cause of sleep deprivation and poor sleep quality for caregivers [52]. This finding however was inconsistent with the insomnia network model of mental health professionals [28] that found “Interference with daytime functioning” (ISI5), “Sleep maintenance” (ISI2), “Noticeability of sleep problems by others” (ISI6), and daytime insomnia symptoms (ISI5, ISI6, ISI7) as influential symptoms, probably due to the working shifts of mental health professionals. The discrepancy between different insomnia network models indicates that the interventions for insomnia symptoms should be individualized for different populations in need. However, this study did not find any clear association between caregivers’ insomnia and the psychiatric diagnoses of patients they cared for in terms of insomnia prevalence and the network structures of insomnia symptoms.

Comorbid psychiatric problems were found to be common in persons with insomnia [53, 54]. Similarly, in this study, more severe depression and anxiety were significantly associated with a higher risk of insomnia. A previous study found that worry about the night-time activities of inpatients could lead to anxiety among the caregivers [52], and if the anxiety and stress continued, the risk of sleep problems might significantly increase. In addition, the bidirectional relationships between insomnia and depression and anxiety have been well documented [53]. On one hand, depression and anxiety could act as risk factors for insomnia; on the other hand, insomnia might manifest as a symptom of depression and anxiety [54]. Further, stressful life events could trigger the development of these problems with insomnia often manifesting first and continue to be persistent [54].

Fatigue was also a significant factor of insomnia among caregivers of psychiatric inpatients. Previous studies found that people suffering from insomnia were more likely to suffer from fatigue than those with any other sleep problems [55, 56]. Fatigue is defined as subjective feelings ranging from tiredness to exhaustion and accompanied by interference with daily functioning [57]. From a psychological perspective, it is associated with stress and other strong emotional experiences that may coexist with depression and anxiety disorders [57]. Caring for patients with severe psychiatric symptoms may lead to high levels of physical, mental and work fatigue among their caregivers [58]. A previous study conducted among insomnia patients found that the relationship between insomnia and fatigue was moderated by comorbid depression [55]. Therefore, it may be that preventing or treating depression can improve fatigue in patients with insomnia.

Consistently, in this study we found that caregivers with insomnia had lower QoL compared to those without insomnia. Insomnia could result in both physical and psychiatric distress [59], which are risk factors of low QoL [60]. In addition, “Sleep dissatisfaction” (ISI4), “Distress caused by the sleep difficulties” (ISI7) and “Interference with daytime functioning” (ISI5) had the strongest negative associations with QoL among caregivers of psychiatric inpatients, which is partly consistent with previous findings in bipolar patients [59]. Disturbed sleep patterns (e.g., insufficient or excessive sleep) were associated with physical and mental health problems such as cardiovascular diseases, diabetes, obesity, depression, suicidal behavior and mortality [61]. Previous studies found that the distress caused by insomnia such as anxiety, depression, irritable feelings and tiredness could lower QoL [62]. Furthermore, persons with insomnia might experience daytime sleepiness and decreased productivity, which could impair social and occupational domains and lower QoL [63]. Targeting these individual symptoms may be beneficial to improve the QoL among caregivers of psychiatric inpatients.

The strengths of this study included the multicenter study design, a large sample size, and use of a novel, sophisticated statistical approach at the symptom level from the perspective of network analysis. However, several limitations should be noted. First, the cross-sectional study design could not ascertain the causal relationships between variables. Second, for logistical reasons, consecutive sampling methods were used, which may limit the representativeness of the study sample. Third, the assessment was based on self-report, therefore, recall bias might occur. Finally, for logistical reasons, the history of psychiatric disorders of caregivers could not be verified as this study based on self-report.

In conclusion, insomnia was common among caregivers of psychiatric inpatients during the COVID-19 pandemic, particularly in those with depression, anxiety and fatigue. Considering the negative impact of insomnia on QoL, regular screening and effective interventions for insomnia should be developed for caregivers of psychiatric inpatients, particularly targeting the central symptoms identified in this study.

### Electronic supplementary material

Below is the link to the electronic supplementary material.

Supplementary Material 1: Supplementary tables and figures related to network models of insomnia symptoms

## Data Availability

The datasets used and/or analyzed during the current study are available from the corresponding author on reasonable request.
