# Supplementary material for: Prevalence of insomnia and its association with quality of life in caregivers of psychiatric inpatients during the COVID-19 pandemic: a network analysis
Source: BMC Psychiatry. 2023 Nov 14;23:837. doi: 10.1186/s12888-023-05194-w (PMC10644468; doi:10.1186/s12888-023-05194-w)
Supplement: Supplementary file 1 — Supplementary Material 1: Supplementary tables and figures related to network models of insomnia symptoms [file 12888_2023_5194_MOESM1_ESM.docx]

**fSupplementary materials**

Figure S1. Network stability of insomnia symptoms among caregivers of psychiatric inpatients

Figure S2. Bootstrapped confidence intervals of edge-weights

Figure S3. Estimation of edge-weights difference by bootstrapped difference test

Table S1. Descriptive information and network centrality indices of insomnia symptoms

| Items | Item content | Mean (SD) | Predictability | EI | Predictability* | EI* |
| --- | --- | --- | --- | --- | --- | --- |
| ISI1 | Severity of sleep onset | 0.57 (0.80) | 0.729 | 0.982 | 0.729 | 0.978 |
| ISI2 | Sleep maintenance | 0.50 (0.77) | 0.744 | 1.094 | 0.744 | 1.085 |
| ISI3 | Early morning wakening problems | 0.59 (0.76) | 0.527 | 0.673 | 0.529 | 0.717 |
| ISI4 | Sleep dissatisfaction | 1.00 (0.91) | 0.479 | 0.650 | 0.479 | 0.650 |
| ISI5 | Interference with daytime functioning | 0.56 (0.80) | 0.675 | 0.963 | 0.675 | 0.949 |
| ISI6 | Noticeability of sleep problems by others | 0.50 (0.79) | 0.683 | 0.927 | 0.683 | 0.915 |
| ISI7 | Distress caused by the sleep difficulties | 0.48 (0.80) | 0.740 | 1.038 | 0.740 | 1.034 |

Notes: EI: Expected Influence; SD: standard deviation; * Adjusted network after controlling for age

Table S2. Edge-weights of QoL and insomnia symptoms

| Items | QoL | ISI1 | ISI2 | ISI3 | ISI4 | ISI5 | ISI6 | ISI7 |
| --- | --- | --- | --- | --- | --- | --- | --- | --- |
| QoL | 0.000 |  |  |  |  |  |  |  |
| ISI1 | -0.008 | 0.000 |  |  |  |  |  |  |
| ISI2 | -0.048 | 0.473 | 0.000 |  |  |  |  |  |
| ISI3 | -0.023 | 0.064 | 0.280 | 0.000 |  |  |  |  |
| ISI4 | -0.175 | 0.165 | 0.041 | 0.156 | 0.000 |  |  |  |
| ISI5 | -0.075 | 0.031 | 0.087 | 0.059 | 0.169 | 0.000 |  |  |
| ISI6 | 0.000 | 0.114 | 0.025 | 0.085 | 0.000 | 0.330 | 0.000 |  |
| ISI7 | -0.120 | 0.126 | 0.165 | 0.016 | 0.061 | 0.252 | 0.368 | 0.000 |

Note: QoL: quality of life

Figure S1. Network stability of insomnia symptoms among caregivers of psychiatric inpatients


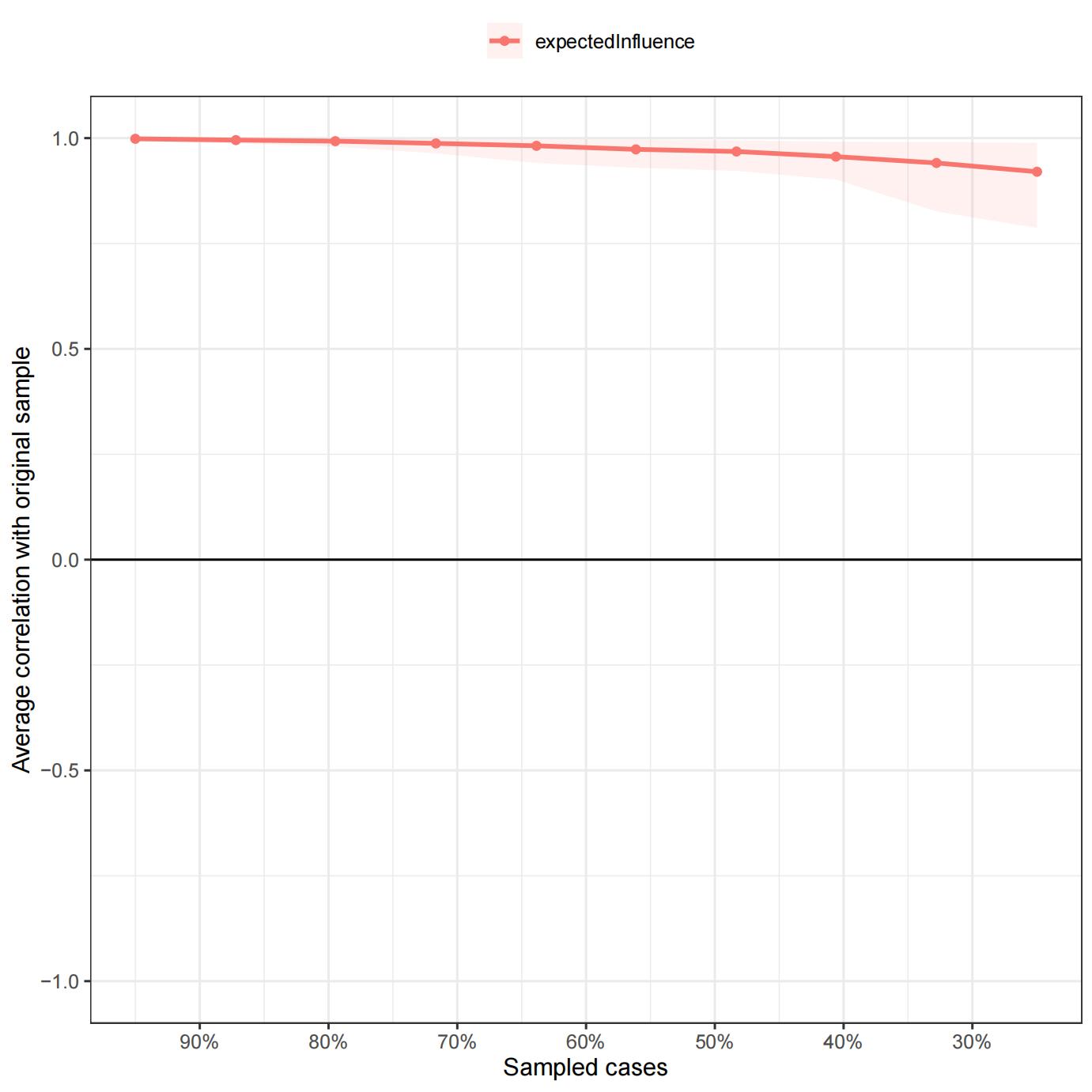


Figure S2. Bootstrapped confidence intervals of edge-weights

**
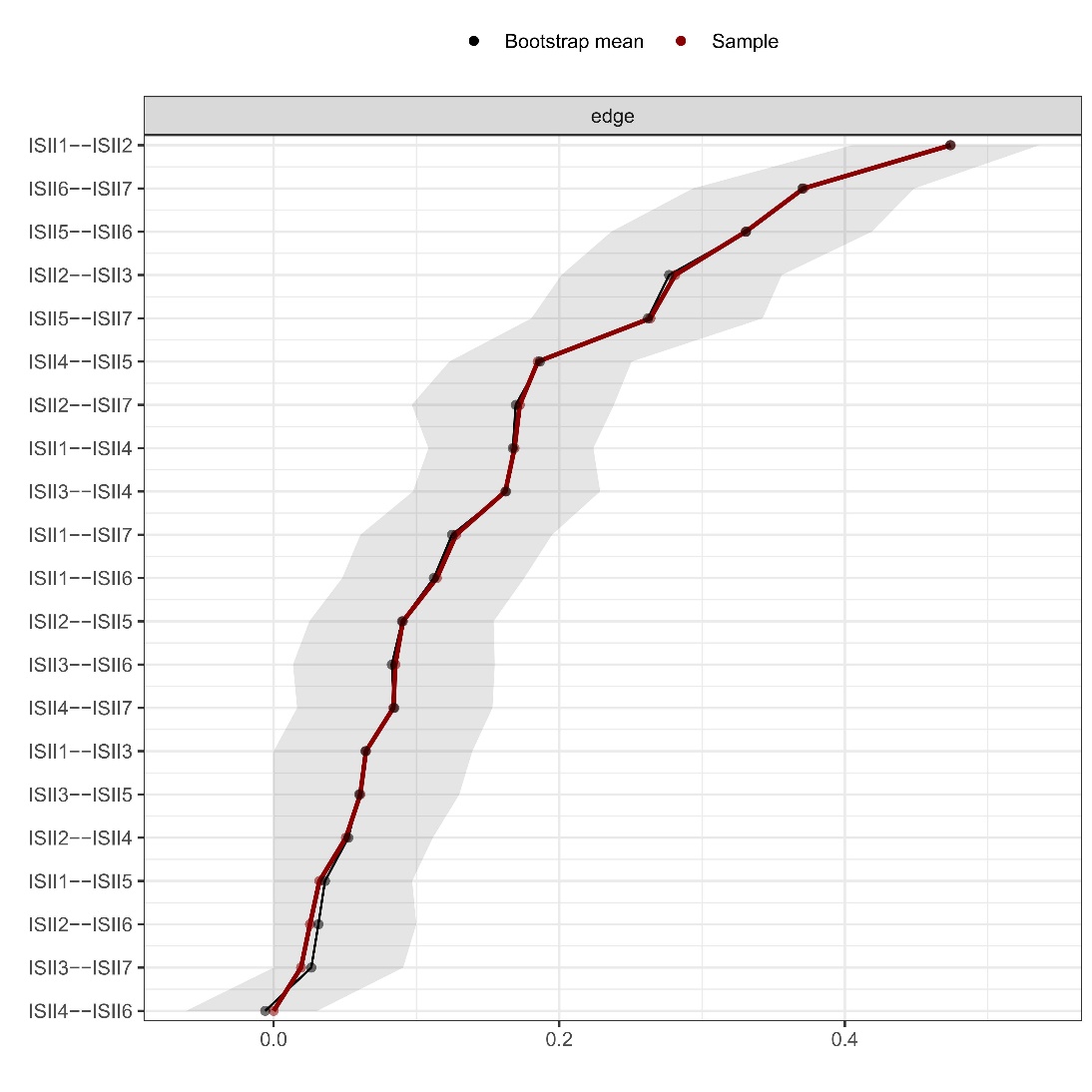
**

Notes: The black dots indicate the values of each edge weight, ordered from the highest to the lowest value. The gray area represents the 95% confidence intervals of edge weights, estimated with the non-parametric bootstrap procedure. (ISI1: Severity of sleep onset; ISI2: Sleep maintenance; ISI3: Early morning wakening problems; ISI4: Sleep dissatisfaction; ISI5: Interference with daytime functioning; ISI6: Noticeability of sleep problems by others; ISI7: Distress caused by the sleep difficulties)

Figure S3. Estimation of edge weights difference by bootstrapped difference test


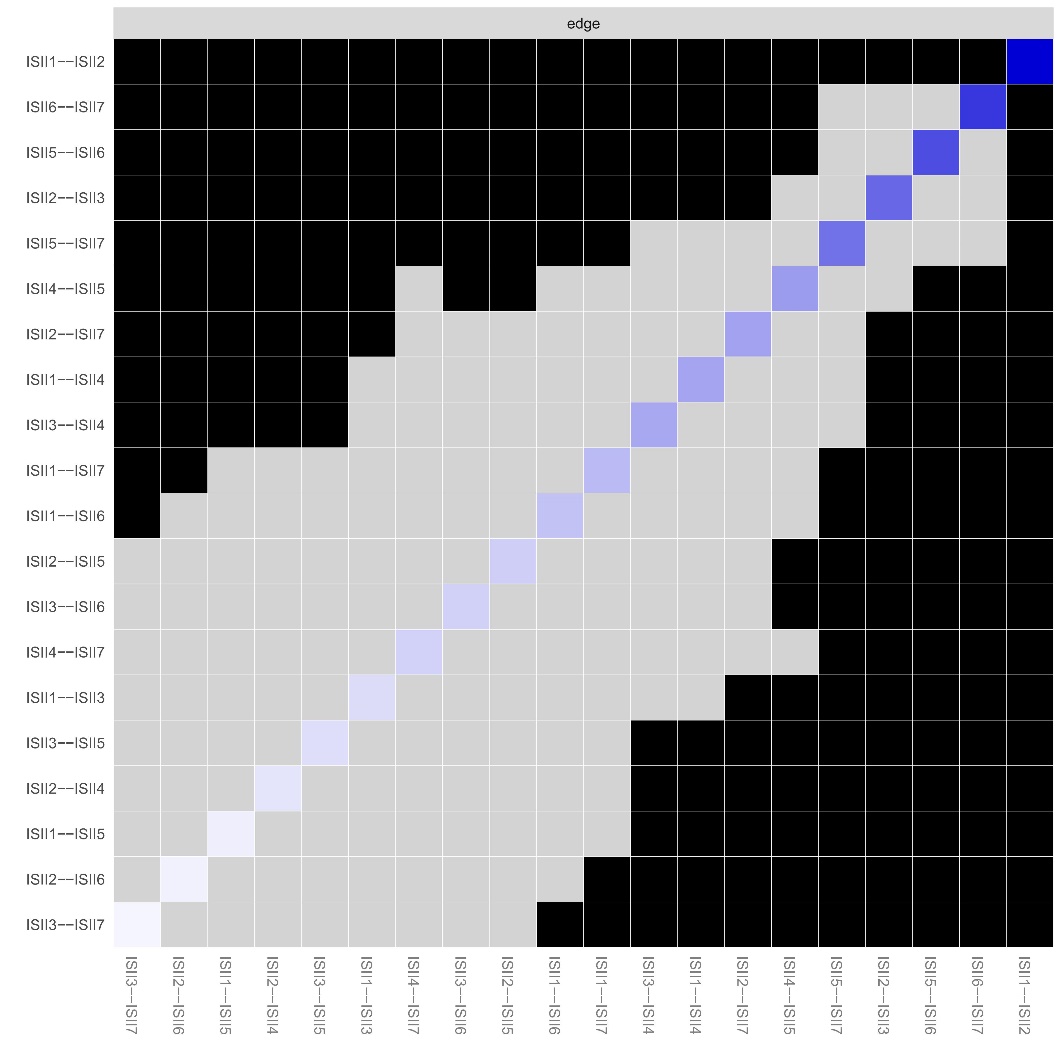


Notes: Gray boxes indicate edges that do not significantly differ from one-another. Black boxes represent edges with significant difference from one another (α = 0.05). Blue boxes in the edge-weight plot indicate positive correlations.
